# Retracted: Antenatal Steroid Therapy for Fetal Lung Maturation and the Subsequent Risk of Childhood Asthma: A Longitudinal Analysis

**DOI:** 10.1155/2016/6212584

**Published:** 2016-10-12

**Authors:** Journal of Pregnancy

Journal of Pregnancy has retracted the article titled “Antenatal Steroid Therapy for Fetal Lung Maturation and the Subsequent Risk of Childhood Asthma: A Longitudinal Analysis” [1]. The article was found to contain a substantial amount of material from the following published article by the same authors: D. Pole, C. A. Mustard, T. To, J. Beyene, and A. C. Allen, “Antenatal steroid therapy for fetal lung maturation: is there an association with childhood asthma?” Journal of Asthma, vol. 46, no. 1, pp. 47–52, 2009. That article was cited in the “Methods” section, but not discussed.
